# Insights into teleost sex determination from the *Seriola dorsalis* genome assembly

**DOI:** 10.1186/s12864-017-4403-1

**Published:** 2018-01-08

**Authors:** Catherine M. Purcell, Arun S. Seetharam, Owyn Snodgrass, Sofia Ortega-García, John R. Hyde, Andrew J. Severin

**Affiliations:** 1 Southwest Fisheries Science Center, National Marine Fisheries Service, 8901 La Jolla Shores Drive, La Jolla, CA 92037 USA; 2 Genome Informatics Facility, Iowa State University, 206 Science I, Ames, IA 50011 USA; 3 Instituto Politécnico Nacional-Centro Interdisciplinario de Ciencias Marinas, Departmento de Pesquerías, Av. Instituto Politécnico Nacional s/n Col. Playa Palo de Santa Rita C.P, 23096 La Paz, Mexico

**Keywords:** California yellowtail, Estradiol, Genomic resources, *hsd17b1*, *Seriola dorsalis*, Sex-determining region

## Abstract

**Background:**

The assembly and annotation of a genome is a valuable resource for a species, with applications ranging from conservation genomics to gene discovery. Genomic resource development is especially important for species in culture, such as the California Yellowtail (*Seriola dorsalis*), the likely candidate for the establishment of commercial offshore aquaculture production in southern California. Genomic resource development for this species will improve the understanding of sex and other phenotypic traits, and allow for rapid increases in genetic improvement for and economic gain in culture production.

**Results:**

We describe the assembly and annotation of the *S. dorsalis* genome, and present resequencing data from 45 male and 45 female wild-caught *S. dorsalis* used to identify a sex-determining region and marker in this species. The genome assembly captured approximately 93% of the total 685 MB genome with an average coverage depth of 180×. Using the assembled genome, resequencing data from the 90 fish were aligned to place boundaries on the sex-determining region. Sex-specific markers were developed based on a female-specific, 61 nucleotide deletion identified in that region. We hypothesize that Estradiol 17-beta-dehydrogenase is the putative sex-determining gene and propose a plausible genetic mechanism for ZW sex determination in *S. dorsalis* involving a female-specific deletion of a transcription factor binding motif that may be targeted by Sox3.

**Conclusions:**

Understanding the mechanism of sex determination and development of assays to determine sex is critical both for management of wild fisheries and for development of efficient and sustainable aquaculture practices. In addition, this genome assembly for *S. dorsalis* will be a substantial resource for a variety of future research applications.

**Electronic supplementary material:**

The online version of this article (10.1186/s12864-017-4403-1) contains supplementary material, which is available to authorized users.

## Background

Aquaculture production has become increasingly important to satisfy seafood and fishery product demands. The fraction of the global seafood market produced using aquaculture has steadily grown, and for the first time in 2014, aquaculture production provided more fish than capture fisheries [1]. Despite this growth, aquaculture development in the U.S. has lagged behind other countries, ranking 17th in aquaculture production while importing greater than 80% of seafood consumed [1, 2]. To date, most U.S.-based finfish aquaculture has focused on the Channel Catfish (*Ictalurus punctatus*), Rainbow Trout (*Oncorhynchus mykiss*), Atlantic Salmon (*Salmo salar*), tilapia (*Oreochromis* and *Tilapia* spp.), and hybrid Striped Bass (*Morone saxatilis x M. chrysops*) [2], however, there is growing interest in broadening the species variety in aquaculture production. The fishes of the genus *Seriola* (*S. dorsalis, S. dumerili, S. lalandi, S. rivoliana, S. quinqueradiata*), collectively known as amberjacks or yellowtail, are of interest due to their high value in the sashimi industry, and are already highly valued in global aquaculture production. While *S. rivoliana* is already in U.S.-based culture, the native California Yellowtail, *Seriola dorsalis*, is a focus for imminent development of offshore aquaculture in southern California and Mexico.

Development of environmentally friendly and economically sustainable aquaculture requires an understanding of the genetic basis of traits that currently limit/enhance development of domestic aquaculture [3]. Genetic resources have been developed and used extensively in agriculture and livestock breeding for decades, but have only more recently been applied to select aquaculture species (e.g. Rainbow Trout, Atlantic Salmon, tilapia, catfish, flounder, Atlantic Cod) [4, 5]. These resources have been used to identify genetic variation underlying phenotypic traits of economic interest for aquaculture production, for example, disease resistance, growth rate, tolerance of environmental stressors, diet/nutrition, reproduction, and general health [5–8]. Methods to develop these resources provide the best possibilities for genetic improvement of broodstock or culture practices [9]. Next generation sequencing (NGS) has revolutionized this area of research through decreasing costs and increasing number of research applications [5], and this has enabled development of genetic resources for a greater number of species [7].

The ability to determine sex is often one of the first characteristics targeted following genomic resource development. In wild fish studies, determining sex from fin clips or plugs of muscle tissue is important for evaluating population composition, sex-biased movements, and stock assessment models [3]. Genetic markers would also allow sex data to be collected from fish sampled non-lethally (e.g., fin clips), and from immature individuals, for which sex may not be determined even with lethal dissection [10]. In aquaculture, economically valuable traits may be linked to sex, such as growth rate, size at maturity, age at sexual maturity, color pattern, fin shape, and even fillet flavor [3, 11–13]. Genetic sex identification would allow aquaculture producers to take advantage of sexual dimorphism, improve broodstock selection efficiency, and accelerate monosex culture development [13]. These economic and conservation-based considerations highlight the importance of understanding sex determination mechanisms and developing sex-linked markers for commercially valuable fish species.

Sex-specific markers have only been identified for a handful of fish species [12] due to remarkable variety in sex determination (SD) mechanisms observed in teleost fish, which can vary in closely related species and even within different populations of the same species [14, 15]. Varied SD modes include chromosomal, polygenic, epigenetic and environmental [16, 17], and within better-studied chromosomal systems, XY (males are the heterogametic sex)/ZW (females are the heterogametic sex), underlying SD mechanisms still vary greatly in teleosts. For example, the XY SD system in Tiger Pufferfish (*Takifugu rubripes*) is controlled by a single nucleotide difference in the anti-Müllerian hormone receptor type II gene (AmHRT2) while the Patagonian Pejerrey (*Odontethes hatcheri*) has a Y-linked duplicated copy of the anti-Müllerian hormone [18]. The Medaka (*Oryzias latipes*) genome contains a Y-linked duplication (DmY) of the Dmrt1 gene [19, 20], and in Rainbow Trout, a Y-linked gene (sdY) encodes a regulatory protein [21]. It is difficult to distinguish sex chromosomes/regions in teleosts, as they are often not heteromorphic due to relatively recent origins or newly emerging/changing master regulator sex-determining genes or genomic regions [22–25]. In some fish, such as the Zebrafish (*Danio rerio*) multiple factors regulate SD that requires a quantitative threshold of gene accumulation that pushes the trait beyond threshold for either sex [16, 26–28]. This implies sex is a fickle phenotype, not consistently determined by any single gene [29].

For *Seriola*, previous research on *S. quinqueradiata* suggested a ZW sex determining system [30, 31], and linkage analyses identified markers associated with sex. However, these regions did not identify sex in other *Seriola* species including *S. dorsalis* (V. Martinez, personal communication; A. Ozaki, personal communication; Purcell, unpublished data). To accelerate growth of yellowtail aquaculture in the U.S., we have worked to begin developing genomic resources for the California Yellowtail in order to identify markers, such as sex-determining markers, that will be advantageous to optimizing culture techniques. In the present study, we describe the assembly and annotation of the *Seriola dorsalis* genome, and present resequencing data from 45 male and 45 female wild-caught *S. dorsalis* used to identify a sex-determining region and marker in this species.

## Methods

### Specimen collection

For genome sequencing, a juvenile *S. dorsalis* (50 days post-hatching) was sampled from Hubbs SeaWorld Research Institute (San Diego, CA) during an aquaculture production run. This individual was humanely euthanized by placing the fish in a bath containing a lethal overdose (a concentration of 800 mg/L) of the anesthetic tricaine methanesulfonate (MS-222). The whole juvenile fish was then placed immediately into RNA*later*® Stabilization Solution (AMBION, Thermo Fisher Scientific, Waltham, MA), stored for 24 h at 4 °C and then frozen at −20 °C until DNA extraction. Mature wild California Yellowtail were sampled from San Diego, California, Cedros Island, Mexico, and La Paz, Mexico. Tissue specimens were acquired via hook and line sampling by private sport anglers or commercial/subsistence fishers aboard various fishing vessels, scientific observers then sampled these specimens shipboard or at landing docks. Biologists from the National Marine Fisheries Service – Southwest Fisheries Science Center or the Instituto Politécnico Nacional-Centro Interdisciplinario de Ciencias Marinas (Mexico) dissected each fish to examine gonadal tissue; if eggs or milt could be identified in the individual fish, the sex was recorded and a genetic sample was collected, ambiguous gonads (e.g. from immature individuals) were not collected as sexed-samples. Gonadal developmental stages in the Yellowtail were not assessed, as this was beyond the scope of this project. From these fish, pieces of muscle tissue, fin clips, or gonadal tissue were placed in 100% ethanol until DNA extraction; the type of tissue varied by sample. Several hundred sexed-specimens were collected from these locations, and 90 of these fish (15 of each sex from each of the three locations, for a total of 45 male and 45 female specimens), were selected to undergo sequencing based on tissue quantity and DNA quality (see below). Sexed-specimens not undergoing sequencing were used to test the accuracy and amplification of the developed sex-specific marker primers.

### DNA extraction

For all specimens, genomic DNA was extracted using the DNeasy Blood and Tissue Kit (Qiagen, Germantown, MD) following the manufacturer’s protocol. Heart and spleen tissue were used from the specimen undergoing genomic sequencing. Muscle, fin, or gonadal tissue was used for the 90 specimens undergoing resequencing and for the additional specimens screened with the sex-specific markers. Purification of extracted genomic DNA was assessed using a NanoDrop ND-1000 with the spectrophotometer absorbance ratio of 260/280 nm, and DNA quantification was performed using a PicoGreen® (Invitrogen, Carlsbad, CA) based assay on the VictorX3™ 2030 (Perkin Elmer, Waltham, MA). Samples prepared for the genomic and re-sequencing applications were sent to the DNA Sequencing Facility at Iowa State University (Ames, Iowa) for library preparation and sequencing.

### DNA sequencing and library insert sizes

Four libraries were prepared for genomic sequencing: three mate-paired (MP) libraries with insert sizes of 2000 bp, 8000 bp, and 12,000 bp and one 300 bp paired-end (PE) library. Each library was run in a single lane (four lanes total) on the Illumina HiSeq 2500 sequencer (Illumina, San Diego, CA). For the resequencing approach, 90 wild-caught samples were indexed and 100-bp PE libraries were run on four lanes of the Illumina HiSeq 2500 resulting in approximately 2× coverage per sample.

### Assembly and annotation of the genome

FASTQ formatted files of paired-end and mate-pair reads generated from the HiSeq2500 in FASTQ format were used for the assembly. Prior to assembly, FastQC (https://www.bioinformatics.babraham.ac.uk/projects/fastqc/) was run to verify the quality of the reads. MaSuRCA assembler (version 2.3.2) [32] was used to assemble the raw data into 98,162 scaffolds and has been deposited at DDBJ/ENA/GenBank under the accession PEQF00000000. To obtain a more reasonable assembly for downstream bioinformatics analyses and for visualization in JBrowse [33], scaffolds were filtered with the following parameters: scaffolds were removed when they contained fewer than 800 bases, or when 90% of the total scaffold length was contained in a different larger scaffold. The scaffolds must also contain a gene or be larger than 10,000 bases. This filtering resulted in 4717 scaffolds remaining in the assembly (bioprojectID PRJNA319656). The scaffolds were then scrutinized for contamination. To identify PhiX contamination (a type of contamination introduced by Illumina’s sequencing kits), the NCBI Reference Sequence: NC_001422.1 was blast queried against the genome assembly, and one scaffold (scaffold_26907) was identified and removed. Blobtools [34] was used to identify another 277 scaffolds (contamination277.txt) that appeared to contain contamination from the phytoplankton *Emiliania huxleyi,* and the number of scaffolds was further reduced. The quality of the final assembly was assessed using BUSCO [35]; this program provides a measure of genome assembly quality by determining the number of conserved single-copy orthologs found within the assembled genome compared to the BUSCO ortholog database.

Utilizing raw RNA-Seq data (if available) is useful in genome assemblies to identify exon/intron boundaries more accurately than methods that rely on assembled transcripts. To assist in annotating this genome, data were used from a concurrent RNA-Seq project on *S. dorsalis* that examined slow- and fast-growing larvae at three early developmental time points (Purcell et al.*,* unpublished data). BRAKER [36] was used to annotate the genome using 547 million 50 bp PE raw RNA-Seq data from that project. The BRAKER pipeline uses GeneMark-ET [37] to perform unsupervised training using the genome file and the RNA-Seq data. After training, GeneMark-ET creates an ab initio gene set, and those gene structures in all introns, that have support from RNA-Seq alignments, are then selected for automated training of AUGUSTUS [38]. After training, AUGUSTUS predicts genes in the input genome file using spliced alignment information from RNA-Seq as extrinsic evidence. RNA-Seq data for this project was also deposited at DDBJ/ENA/GenBank under the BioProject ID: PRJNA339646.

### Alignment of sexed-wild yellowtail and single nucleotide polymorphism (SNP) calling

Raw data from the sequences of the 90 sexed-wild caught yellowtail were quality checked using FastQC [39]. Raw reads were aligned to the assembled genome using BWA-MEM [40].The aligned bam files were prepared for SNP calling by GATK [41]; this included coordinate sorting, cleaning, duplicate marking, adding of read groups (http://broadinstitute.github.io/picard) and SNP/Indel realignment. GATK was then run to call SNPs and InDels on the combined alignment files.

### Identification of the sex-determining region (SDR)

A genome-wide association study (GWAS) using a Generalized Linear Model (GLM) was performed in TASSEL (version 5) [42] to identify genomic regions significantly correlated with the sex phenotype. SNPs were imputed using Euclidean distance by mean with the five nearest neighbors. The kinship matrix was calculated using a centered Identity By State (IBS) with a maximum number of six alleles. This required joining three datasets (imputed SNPs, traits and kinship matrix) using the union join command under the data menu. The *p*-value cutoff was set to 1e-3 and 1000 permutations were selected. TASSEL could not handle the full SNP dataset, so SNP subsets were generated using vcf-subset.py (https://github.com/ISUgenomics/common_scripts/blob/master/vcf-subset.py), which takes a random SNP from every 5000 base interval. GLM was then repeated for all SNPs on chromosomes where multiple co-linear SNPS had a significant correlation. For comparison, the sex marker in *Seriola quinqueradiata (*ssr263g21) [31] was also mapped to the *S. dorsalis* genome using GMAP [43].

The number of heterozygous SNPs in the SDR were compared between males and females. A SNP was considered heterozygous if the allelic ratio for all individuals in each population was between 0.4 and 0.6. If *S. dorsalis* exhibits a ZW type of sex-determination, as seen in *S. quinqueradiata* [30, 31], a higher number of heterozygous SNPs would be expected in females, while a higher number would be expected in males if the system is XY. As a control, for any identified SDRs, the same region on five different scaffolds (not containing the SDR) were compared across the three sampling locations (San Diego, Cedros Island, and La Paz) between males and females; the expectation was that no significant male/female differences in these other regions would be identified.

### Sex-determining marker development

All insertions/deletions (InDels) greater than 40 nucleotides in the SDR were identified. Two sets of primers, SdorDel01 and SdorDel02, were designed to span across a region of interest using Primer3 [44] (Table 1). These primers were tested for consistent amplification and accuracy as a sex-determining marker on the 90 specimens used for sequencing in this study, and in another set of 212 known-sex specimens. Polymerase chain reactions (PCRs) were conducted in 25 μl volumes containing approximately 10–20 ng template DNA in a reaction containing 67 mM Tris-HCl pH 8.8, 16.6 mM (NH_4_)_2_SO_4_, 10 mM β-mercapto-ethanol, 2 mM MgCl_2_, 800 μM dNTPs, 0.5 mg/ml BSA, 0.15–0.3 μM forward primer, 0.15–0.3 μM reverse primer, and 0.25 units *Taq* DNA polymerase (New England Biolabs, Ipswich, MA). Thermal cycling parameters were as follows: initial denaturing at 94 °C for 4 min., 40 cycles of 94 °C for 30 s, annealing at 53 °C for 55 s, extension at 72 °C for 55 s, and a final extension step at 72 °C for 5 min. PCR products were electrophoresed at 78 V for 40 (SDorDel02) and 60 (SDorDel01) minutes through a 2% agarose gel, with a 100-bp size standard (Thermo Fisher Scientific, Waltham, MA) run in a lane adjacent to PCR products, Ethidium bromide stained gels were visualized and digitally acquired on an UV light box using Enduro™ GDS Touch and recorded using the Labnet Enduro Gel Documentation System Image Acquisition Software (v. 1.3.1218.0) (Labnet, Edison, NJ).

**Table 1:** Primer names, sequences, melting temperatures (Tm), lengths, and PCR fragment sizes

Primer Name	Sequence	Tm (°C)	Length (bp)	Fragment Size (without/with deletion)
SdorDel01-F	AATTCATCCAAACCCAGCAG	59.9	20	452 bp/391 bp
SdorDel01-R	GGTCTTGTCAACTGCGATCA	59.8	20	
SdorDel02-F	TGACAACAAGGCAACAGGAG	59.9	20	282 bp/221 bp
SdorDel02-R	TTGGCCTTTCTTTTGACCAC	60.1	20	

### Identification of genes related to sex-determination

Regions containing genes known to be involved in sex determination and differentiation are a good starting place for identifying candidate regions involved in sex determination for non-model organisms. These genes were identified from the literature: *hsd17b3 cyp19a1a, hsd17b1*, *foxl2, dmrt1*, *sox9*, *sox3*, *sf1* and *amh* [16, 20, 45–47] and the corresponding genes in *S. dorsalis* were identified using the annotation in the GFF file. Orthologs for the *S. dorsalis* genes were identified using a reciprocal best BLAST to six fish genomes downloaded from NCBI (0.82): *Danio rerio, Lepisosteus oculatus, Oreochromis niloticus, Oryzias latipes, Takifugu rubripes* and *Xiphophorus maculatus*. The BLAST results were filtered requiring at least 50% coverage of the *S. dorsalis* gene to be considered orthologous (Additional file 1: Table S1)*.*

### Identification of potential binding motifs

Identification of transcription factor binding motifs can be challenging due to their short sequence lengths, which can lead to false positives, and due to the limited number of known transcription factor binding motifs contained in databases (e.g. Jaspar) [48]. Phylogenetic footprinting was used in the analysis; this approach uses conserved sequences identified between orthologous upstream regions to improve odds of a true positive discovery. Following identification of the *hsd17b1* (Estradiol) gene (see results), a 3000 base nucleotide sequence upstream of this gene was extracted from the *S. dorsalis* genome and from the six fish genomes downloaded from NCBI (Estradialupstream3000.fasta); this was performed using the gff2fasta.pl script (https://github.com/ISUgenomics/common_scripts/blob/master/gff2fasta.pl). This upstream region from the reciprocal best blast hits (RBBH) analysis was scanned for conserved motifs using MEME (Motif-based sequence analysis tools) [49]. MEME was performed on these sequences to identify 100 potential motifs using the following parameters: -dna -mod anr -revcomp -p 16 -nmotifs 100. A curated database of experimentally defined transcription factor binding sites, Jaspar [48] was used to identify potential binding motifs, and the corresponding transcription factor, to determine if any binding sites were related to sex hormone regulation and sex determination.

### Raw data

All raw data can be downloaded from NCBI under the bioprojectID PRJNA319656.

## Results

### Assembly and annotation statistics

Approximately 1.2 billion reads were generated from the four library preparations. Based on the estimated genome size of 685 MB for *Seriola lalandi* (C-value of 0.70) [50], the sequencing coverage is 180× for the *S. dorsalis* genome. The MaSuRCA assembly resulted in 4439 scaffolds based on 23,003 contigs with N50 s of 1,491,863 bases and 139,330 bases, respectively, and the longest scaffold is 8,096,577 bases. Total genomic content is 653,009,476 bases, which represents 93% of the estimated genome size, with approximately 13 million gaps or ambiguous bases (Ns). The completeness of the assembly is very high based on the BUSCO assessment; this genome contains 2848/3023 BUSCO groups and is only missing 175 of the Eukaryotic orthologues.

There were 49,784 transcripts corresponding to 45,251 genes, and of these, 8155 genes had RBBH to orthologs covering at least 50% of the gene sequence in *Seriola* and in six other fish species (*Danio rerio, Lepisosteus oculatus, Oreochromis niloticus, Oryzias latipes, Takifugu rubripes* and *Xiphophorus maculatus*) (Additional file 1). An additional 22,011 genes had RBBH to at least one other fish species, and there were 27,218 genes that had a unidirectional blast hit to one of the six fish species, that covered at least 90% of the gene length. Most gene models (42,847) have evidence supported by RNA-Seq data or contain a PFAM (protein family) domain.

### Identification of the sex-determining region

**Fig. 1:**
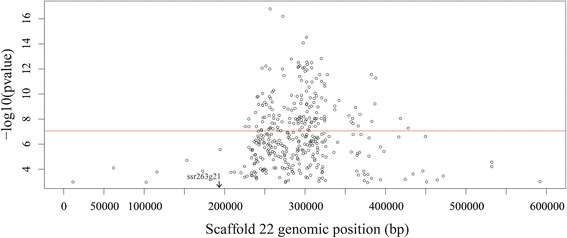
Negative log 10 plot of p-value versus genomic location (bp) on Scaffold_22. *P*-values were determined via GWAS of sex phenotype using TASSEL. A black arrow indicates the position of the *S. quinqueradiata* sex marker (ssr263g21). A red line is drawn at a *p*-value of 1e^−7^ to show significant SNPs in the SDR

The resequencing approach resulted in an average coverage depth of 1.9X for each of the 90 individual fish. From these sequences, GATK called 7,684,767 SNPs and InDels (Additional file 2), of which 7,484,110 were biallelic. From the 7.4 million biallelic SNPs inputted into TASSEL, the GLM analysis used a subset of 375,904 SNPs that spanned across all scaffolds. This GLM analysis indicated that a region on scaffold 22 was strongly correlated to sex (Additional file 3). The GLM repeated for the SNPs on this scaffold revealed that the strong correlation to SD occurred between the nucleotide positions of 231,000 and 320,000 on scaffold 22 (Fig. 1). A negative log 10 *p*-value of nearly 17 for one of the tested SNPs, and the number of significantly linked SNPs, provides strong evidence that this region is correlated with the sex phenotype, and likely represents the SDR for *S. dorsalis*. Additionally, the sex marker identified in *Seriola quinqueradiata (*ssr263g21) [31] mapped to scaffold_22 base 194,817–194,840 in the *S. dorsalis* genome. This is ~36,000 bp upstream of the significantly correlated region in *S. dorsalis*.

### ZW sex determination in *S. dorsalis*

In each sampling location, the ratio of heterozygous SNPs in females was on average 4.9 times higher in the SDR, whereas the ratios in the random genomic locations of the same size were not different between the sexes (Table 2). This heterozygosity pattern indicated a ZW mode of sex-determination within the SDR identified above.

**Table 2:** Numbers of heterozygous SNPs in the SDR versus random genomic regions of the same size

Population	Heterozygous SNP Counts		Ratio	Genomic Region
	Female	Male	(Female/Male)	
CI	90	20	**4.5**	SDR
SD	106	27	**3.9**	SDR
LP	123	20	**6.2**	SDR
LP	140	141	1.0	Random
LP	129	107	1.2	Random
LP	82	91	0.9	Random
LP	261	226	1.2	Random
SD	161	142	1.1	Random
SD	123	97	1.3	Random
SD	101	82	1.2	Random
SD	58	66	0.9	Random
SD	238	263	0.9	Random
CI	146	170	0.9	Random
CI	113	131	0.9	Random
CI	84	91	0.9	Random
CI	55	65	0.8	Random
CI	262	238	1.1	Random

*SDR* Sex Determining Region, *CI* Cedros Island, *SD* San Diego, *LP* La Paz

Bold numbers reflect F/M SNP ratios detected in the SDR

### Sex-determining marker development

**Fig. 2:**
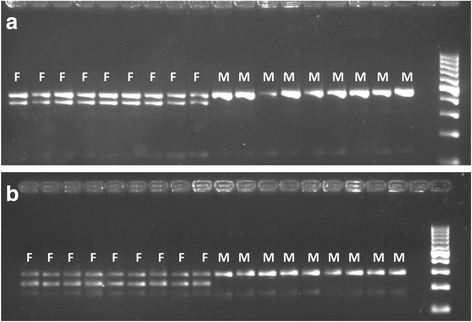
The ethidium bromide stained gel image of the PCR products from the sex-specific genetic markers, SDorDel01 (Gel **a**) and SDorDel02 (Gel **b**) run with nine female (F) and nine male (M) *S. dorsalis* specimens, with a 100-bp ladder in each gel for reference

**Fig. 3:**
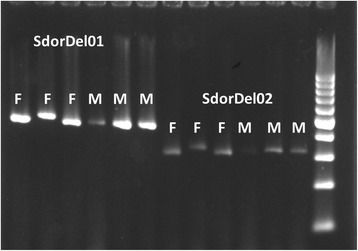
The ethidium bromide stained gel image of the PCR products from the sex-specific genetic markers, SDorDel01 and SDorDel02 run with three female (F) and three male (M) *S. rivoliana* specimens, with a 100-bp ladder for reference

After searching the SDR in *S. dorsalis* for insertions or deletions greater than 40 base pairs, only one 61-base deletion was identified. This deletion was heterozygous in females and not present in males, making it consistent with the ZW sex determination model for *Seriola*. This deletion is located on scaffold 22 at position 246,495 with the following sequence: CGTTCATGATTACTACTTTTACACAAATTTACACAAAAGACATCTGTACCAAAGAACAAAA. The developed primers, SdorDel01 (452 bp) and SdorDel02 (282 bp) consistently amplified and revealed sex-specific patterns on agarose electrophoresis gels (Fig. 2). Heterozygous females displayed two bands, both with and without the 61 bp deletion, while males only exhibited the larger band (without the deletion). These primers were tested on an additional 212 sexed yellowtail and accurately predicted sex in 94% of the individuals. It is unclear whether the disagreement between genetic and phenotypic sex in the remaining 6% of fish (12 individuals) is due to variation in the genomic region that led to poor primer binding, or if the individuals were mis-sexed or mis-labeled upon collection. The two sex-specific primers were also tested for a small number of *S. rivoliana* specimens (*n* = 6), however, the markers failed to distinguish sex for these individuals (Fig. 3).

### Characterizing the sex-determining region

In *S. dorsalis*, there are only seven gene models in the SDR of which only four have known functions: *hsd17b1*: Estradiol 17-beta-dehydrogenase 1, Coenzyme Q-binding protein coq10 homolog, Complement C1q-like protein 2, arf1: ADP-ribosylation factor 1. These genes correspond to Sedor.G00005096, Sedor.G00005097, Sedor.G00005098 and Sedor.G0000599, respectively. The Estradiol 17-beta-dehydrogenase 1 gene (*hsd17b1*) was identified as the most promising sex determing gene within the SDR. This gene (Sedor.G00005096) is present in males and females, but no SNPs were contained within the gene that would suggest a genetic mechanism linked to ZW SD. However, the 61 base deletion (present only in females) was found 954 bases upstream of this gene, indicating this deletion is present only in the W chromosome. One possible hypothesis as to how this deletion might have a role in SD for *S. dorsalis* is if it contained a transcription factor binding motif that suppresses *hsd17b1*.

This was tested with a MEME analysis of the 3000 base nucleotide sequence upstream of this gene in *S. dorsalis* and from the six other fish genomes resulting in several motifs with high levels of significance. One motif in *S. dorsalis* (GTCTTTTGTTCTTTG) overlapped with the deletion on the negative strand; it was found in all seven species and in a similar position upstream of the estradiol gene in five of the seven species, suggesting that it is conserved. After searching for transcription factor binding sites contained in this motif, the Jaspar database revealed binding sites for the following male sex-determining genes: *sry*, *sox9*, and *sox3* with relative scores of 84%, 87%, and 97%, respectively. We caution the reader that while we are able to report the results of a deletion upstream of a gene contained in the pathway involved in sex hormone production and that we can show there are conserved binding motifs in the deletion found on the W chromosome for known male sex determining genes, without further experimental evidence, we cannot confirm our hypothesis.

**Fig. 4:**
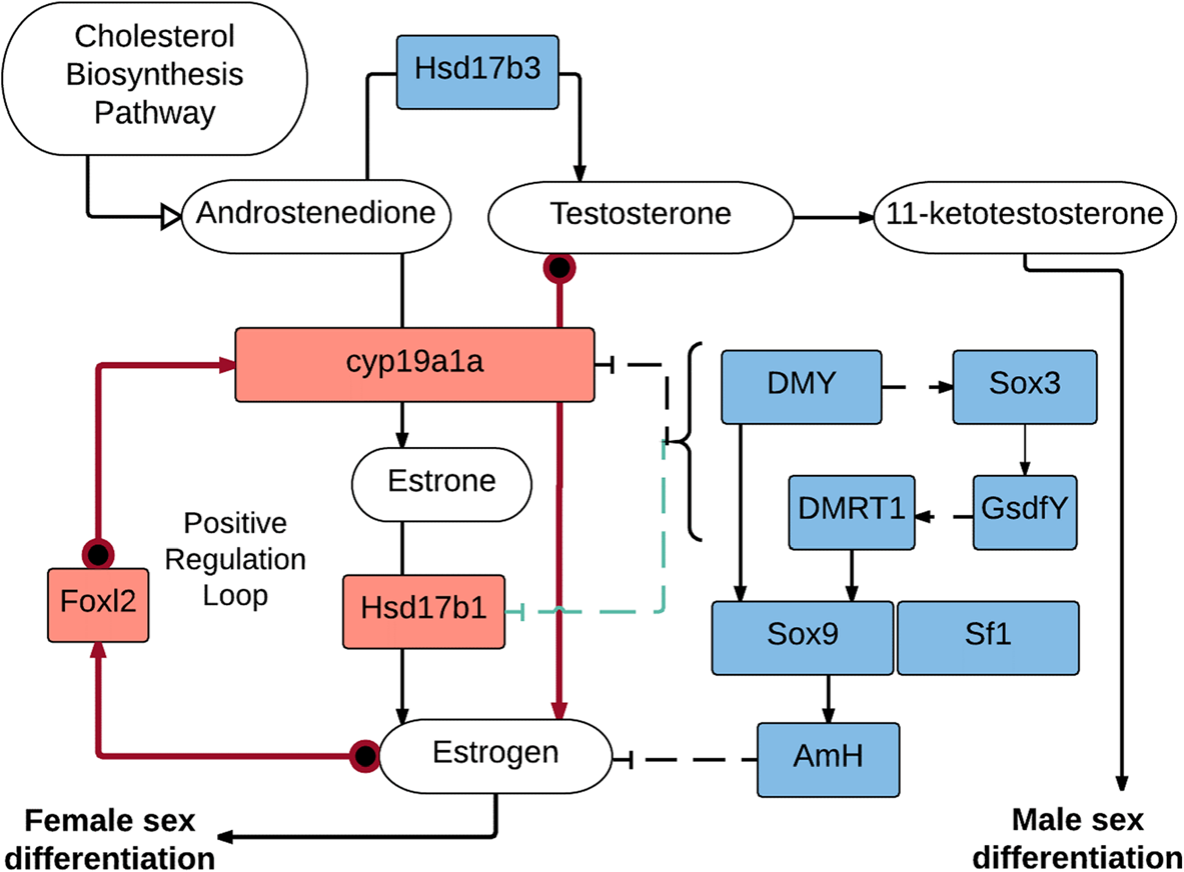
Our interpretation of the sex determination pathway based on the literature. Genes are boxes, gene products are ovals. Genes involved in female and male sex determination are colored in red and blue, respectively. Solid lines indicate known protein interactions while dashed lines indicate hypothesized interactions. Stops (|--) indicate inhibition. The red arrows correspond to the positive regulation loop that drives female sex determination

To gain a better understanding of potential genetic mechanisms of sex determination in *S. dorsalis*, the molecular pathways for sex steroid biosynthesis and genes known to be involved in sex determination were drawn (Fig. 4), based on our literature review and includes models of gene activation, inactivation, protein interactions, and feedback loops. Genes involved in male sex steroid biosynthesis and sex determination include *hsd17b3*, *dmrt1, sox9*, *sox3*, *sf1*, and *amh*. While genes involved in female sex steroid biosynthesis that form a positive regulatory loop leading to female sex differentiation are: *cyp19a1a*, *foxl2* and *hsd17b1* [47, 51].

## Discussion

High throughput sequencing was used to developing genomic resources for *S. dorsalis* in this project. Our assembly and annotation of the California Yellowtail, *S. dorsalis*, is of high quality capturing approximately 93% of the total genome and identifying over 94% of the BUSCO orthologues comparable to other recently published fish genomes [52, 53]. A well-assembled and well-annotated genome is a major contribution for researchers interested in a variety of research questions [54], and this genome will be a powerful resource for additional genomic, transcriptomic, and epigenetic work in California Yellowtail and in other *Seriola* species. Given the importance of *S. dorsalis* and other *Seriola* species for aquaculture production, this genomic resource will be important in accelerating maker-assisted selective breeding programs, and in improving economically relevant culture traits, such as disease resistance, feed acceptance, thermal tolerance, and sex-based characteristics. This genome assembly also enables further investigation of teleost evolution, and the evolution of sex determination, which has proven to be a complex and highly variable trait in fish [13].

Using the aligned resequencing data from the sexed 90 wild-caught fish, boundaries were successfully placed on the SDR and sex-specific makers were developed based on the identified female-specific deletion. The SDR in *S. dorsalis* is consistent with the known sex marker in *S. quinqueradiata (*ssr263g21) [31], which maps just upstream of this region: scaffold 22 at 194817–194840 base pairs (linkage group 12) [30]. However, the marker reported in that study lies just outside of the SDR and has not worked in distinguishing sex in other *Seriola* species (Ozaki, pers. com). Similarly, markers identified in this study did not work in identifying sex in a small number of *S. rivoliana* specimens, although the effectiveness in other *Seriola* species has not yet been tested. It is not surprising that there is difficulty in transferring SD markers among the *Seriola* species. It has been reported that shifts in teleost sex-determining modes are ‘evolutionarily frequent’, even among family-level and genus-level species [55]. Tilapia are one of the most widely known examples of this phenomenon. The mode of sex determination (XX-XY/ZW-ZZ) and the genomic regions associated with SD markers (LG 1 vs. LG 3) differed even among closely related Oreochromis species [56, 57]. Although the SD markers for *S. quinqueradiata* and *S. dorsalis* map relatively closely together, the mechanism that controls the SD pathway may be completely different between these species. As genomic sequencing data are rapidly growing for all *Seriola* species, a future study will be able to examine SD regions among these fish.

The resequencing data also identified that the Estradiol gene (*hsd17b1*) is contained in the SDR; this gene is involved in the female steroid biosynthesis pathway leading to estrogen production [47]. Interestingly, the female-specific 61-nucleotide deletion was found just upstream of this sex steroid biosynthesis gene. The similar position of this motif upstream of the estradiol gene in four of the six species suggests it is well conserved. With this motif matching most closely to transcription factor binding sites associated with male sex determining genes, taken together, these data suggest that estradiol production is suppressed by *sox3*, *sox9* or *sry* with *sox3* being the most likely candidate based on its relatively high Jaspar score.

It is our hypothesis that the large deletion upstream of the estradiol gene (*hsd17b1*) disrupts a silencer motif bound by *sox3* and thereby increases estrogen production leading to the female phenotype. Crispr/Cas9 is the logical choice to confirm this hypothesis in follow-up studies, as it has been used to test similar hypotheses in tilapia by creating deletions in the *foxl2* and *dmrt1* genes [58]. However, experimental studies to test this hypothesis in *S. dorsalis* are beyond the scope of the present study. Insertions or deletions located in a promoter region upstream of a putative master sex-determining gene have been described in other fish species [3]. In Sablefish, for example, sex-specific insertions upstream of the *gsdf* gene were reported [3]. While Rondeau et al. [3] could not conclusively state that *gsdf* is the master sex gene for this species, this gene is evolutionarily conserved and has been reported as a master sex gene in other species, which strongly suggests a similar role in Sablefish.

Although the exact mechanisms for regulation in the sex determination pathways remain unclear, there are examples of “master” SD genes that alter or interfere with upstream regulatory elements. Master SD genes may govern the regulatory networks involved in sex determination, and these genes typically fall under the categories of steroidogenic enzymes, sex steroid receptors, transcription factors, and growth factors [11]. Certain genes that are part of the regulatory network appear to evolve repeatedly into master SD genes, such as *sox3*, *dmrt1*, and *tgf-b* [59]. Although it has not yet been proven, there may be constraints on which genes can become ‘masters’ [16, 31], and some likely candidates (e.g., *foxl2*, *sox9*, *sox8*, and *wnt4*) have not demonstrated the same ability to evolve into master SD genes [59]. Additionally, it has only more recently been demonstrated that downstream networks involved in SD also exhibit some degree of flexibility [59].

While there has been considerable progress in understanding the master gene networks, there are many master genes that have only recently been discovered and their networks are still being explored [59]. There is strong support for both positive [11] and negative [51, 60] regulation between the male and female SD pathways. In non-mammalian vertebrates there are multiple examples of female sex determination depending on a positive feedback loop involving stimulation of *cyp19a1* and the transcription factor, *foxl2*; in males, sex determination depends on inhibition of *cyp19a1* through *dmrt1* upregulation [11]. An example of this mechanism in teleost fish can be found in the European Seabass (*Dicentrarchus labrax*) where the *cyp19a1* promoter is hypermethylated in males compared to females. This methylation causes repression of transcription, and therefore inhibition of the *cyp19a1* gene, preventing ovarian differentiation [11]. In another example in the Half-Smooth Tongue Sole (*Cynoglossus semilaevis*), which exhibits a ZZ/ZW sex determination system, *dmrt1* is expressed in the male sex-determination pathway, however in ZW females, the *dmrt1* promotor was hypermethylated and silenced [14, 61]. Examples of positive regulation can be found in the African clawed frog (*Xenopus laevis*) and in two medaka species, *Oryzias luzonesis* and *O. dancena*. Sex determination in *Xenopus laevis*, involves the DM-W gene, essentially a partial duplication of the *dmrt1* gene containing the DNA binding C-terminal domain but not the transcriptional activating N-terminal domain. DM-W acts on sex determination by competing for the upstream activating element that *dmrt1* targets [62]. In *Oryzias luzonesis*, mutations in a conserved motif increased expression of Gsdf [63] and a cis-regulatory element in *Oryzias dancena* has been shown to upregulate sox3 expression [46].

Clearly, upstream regulatory elements are key players in determining sex. In *S. dorsalis*, we hypothesize that the deletion upstream of the estradiol gene (*hsd17b1*) may function to release the silencing motif of *sox3*, a known master SD gene [59]; this then could lead to increased transcription of estradiol and increased estrogen production, ultimately resulting in the female phenotype (Fig. 4). However, our speculation will need to be followed up by lab experiments, potentially with CRISPR to confirm. If this deletion is the underlying genetic mechanism for SD, it will be interesting to discover whether this is also true for all of the *Seriola* species. For *S. quinqueradiata*, Koyama et al. [31] did not find any known SD genes near the SDR detected in their study, although it is difficult to determine this conclusively without genomic sequences of this region, which were not available at the time of their study. The sex-markers developed in this study did not detect the deletion in the few tested *S. rivoliana* specimens; however, this is unlikely due to mutations in the primer-binding site as the products were of the appropriate size. Ongoing sequencing efforts for *S. rivoliana*, *S. quinqueradiata*, and other *Seriola* species should soon uncover the variation in the SDR among these fish, and reveal the ubiquity of the upstream regulatory elements and master sex-determining genes for this genus of fish.

## Conclusions

As a greater number of fish genomes are sequenced, it is possible (even likely) that all genes involved in regulation of sex steroids (Fig. 4) will be discovered to have been co-opted or disrupted to become a “master-regulating” SD gene, given the variety of genetic mechanisms detected so far in teleosts [46]. In addition, more genes like *gsdfy*, outside of the known steroid biosynthesis pathway and currently not known to be related to sex-determination, will be identified and found to affect the quantitative threshold toward either male or female phenotypes or affecting population sex ratios [21]. Sex determination remains a complex competition between protein expression and protein regulation through transcription factor binding sites in the sex-steroid biosynthesis pathway. Fortunately, sequencing costs continue to drop and knowledge obtained from even low coverage resequencing, as demonstrated here, can gain significant insight into the mechanics of sex determination in teleosts. This genome assembly for *S. dorsalis* will be a substantial resource for a variety of research applications such as population genomics, functional genomics, translational studies, and epigenetic research in wild and cultured populations of *S. dorsalis* and other *Seriola* species. The understanding of sex and other phenotypic traits will be improved through this genomic resource development and help to accelerate the rate of genetic improvement in these cultured species [64].

## Additional files

Additional file 1: BLAST results of orthologous sex-determining genes (from literature) with at least 50% coverage of the *S. dorsalis* gene. (DOCX 14 kb)

Additional file 2: SNP and InDels calls (VCF file) for scaffold containing sex determining region. (VCF 35715 kb)

Additional file 3: Tassel significance output for GWAS analysis for sex phenotype. (TXT 52 kb)

## Abbreviations

BLAST: Basic Local Alignment Search Tool
BP: Base pair(s)
BUSCO: Benchmarking Universal Single-Copy Orthologs
GLM: Generalized linear model
GWAS: Genome-wide association study
IBS: Identity by state
INDELS: Insertions/deletions
MP: Mate-paired
MS-222: Tricaine methanesulfonate
NGS: Next generation sequencing
Ns: ambiguous bases
PCR: Polymerase chain reaction
PE: Paired-end
PFAM: Protein family
RBBH: Reciprocal best-blast hits
SD: Sex determination
SDR: Sex-determining region
