# Supplementary material for: Insights into teleost sex determination from the Seriola dorsalis genome assembly
Source: BMC Genomics. 2018 Jan 8;19:31. doi: 10.1186/s12864-017-4403-1 (PMC5759298; doi:10.1186/s12864-017-4403-1)
Supplement: Supplementary file 1 — BLAST results of orthologous sex-determining genes (from literature) with at least 50% coverage of the S. dorsalis gene. (DOCX 14 kb) [file 12864_2017_4403_MOESM1_ESM.docx]

Table S1. BLAST results of orthologous sex-determining genes (from literature) with at least 50% coverage of the *S. dorsalis* gene.

| ***S dorsalis*** | ***D rerio*** | ***L oculatus*** | ***O Nicoticus*** | **Gene** |
| --- | --- | --- | --- | --- |
| **Sedor.T00038134.1** | blank | blank | ENSONIT00000024731 | SOX9 |
| **Sedor.T00021391.1** | ENSDART00000075617 | ENSLOCT00000021635 | ENSONIT00000026187 | SOX3 |
| **Sedor.T00016532.1** | ENSDART00000005676 | ENSLOCT00000015413 | ENSONIT00000006622 | SOX9 |
| **Sedor.T00001825.1** | ENSDART00000163381 | ENSLOCT00000018695 | ENSONIT00000013584 | SF1 |
| **Sedor.T00005096.1** | ENSDART00000020376 | ENSLOCT00000015385 | ENSONIT00000001507 | HSD17B1 |
| **Sedor.T00008688.1** | ENSDART00000130428 | ENSLOCT00000014125 | ENSONIT00000017864 | DMRT1 |
| **Sedor.T00014291.1** | ENSDART00000013803 | ENSLOCT00000007073 | ENSONIT00000006022 | AMH |
| **Sedor.T00025798.1** | ENSDART00000055743 | ENSLOCT00000006044 | ENSONIT00000020324 | SF1 |
| **Sedor.T00026150.1** | ENSDART00000129828 | ENSLOCT00000016432 | ENSONIT00000000198 | CYP19a1 |
| **Sedor.T00028875.1** | ENSDART00000149610 | ENSLOCT00000011273 | ENSONIT00000019079 | HSD17B3 |
| **Sedor.T00035285.1** | ENSDART00000061828 | ENSLOCT00000021531 | ENSONIT00000026114 | FOXL2 |
| **Sedor.T00000256.1** | ENSDART00000020616 | blank | blank | FOXL2 |

| ***S dorsalis*** | ***O latipes*** | ***T rubrupes*** | ***X maculatus*** | **Gene** |
| --- | --- | --- | --- | --- |
| **Sedor.T00038134.1** | blank | ENSTRUT00000044097 | ENSXMAT00000017399 | SOX9 |
| **Sedor.T00021391.1** | ENSORLT00000002213 | blank | ENSXMAT00000019592 | SOX3 |
| **Sedor.T00016532.1** | ENSORLT00000009986 | ENSTRUT00000047756 | ENSXMAT00000012170 | SOX9 |
| **Sedor.T00001825.1** | ENSORLT00000015944 | ENSTRUT00000012143 | ENSXMAT00000015435 | SF1 |
| **Sedor.T00005096.1** | ENSORLT00000005207 | ENSTRUT00000038976 | ENSXMAT00000009153 | HSD17B1 |
| **Sedor.T00008688.1** | ENSORLT00000025781 | ENSTRUT00000040266 | ENSXMAT00000015647 | DMRT1 |
| **Sedor.T00014291.1** | ENSORLT00000006359 | ENSTRUT00000045920 | ENSXMAT00000012886 | AMH |
| **Sedor.T00025798.1** | ENSORLT00000011264 | ENSTRUT00000034079 | ENSXMAT00000015229 | SF1 |
| **Sedor.T00026150.1** | ENSORLT00000003689 | ENSTRUT00000043649 | ENSXMAT00000002205 | CYP19a1 |
| **Sedor.T00028875.1** | ENSORLT00000005354 | ENSTRUT00000037665 | ENSXMAT00000015851 | HSD17B3 |
| **Sedor.T00035285.1** | ENSORLT00000025059 | ENSTRUT00000019818 | ENSXMAT00000020294 | FOXL2 |
| **Sedor.T00000256.1** | ENSORLT00000006847 | ENSTRUT00000025441 | blank | FOXL2 |
